# 1224. *In vitro* Activities of Ceftaroline and Comparator Agents against Gram-positive Bacterial Pathogens Causing Blood Stream Infections in a Global Population: ATLAS Surveillance Program 2012-2019

**DOI:** 10.1093/ofid/ofab466.1416

**Published:** 2021-12-04

**Authors:** Meredith Hackel, Gregory Stone, Daniel F Sahm

**Affiliations:** 1 IHMA, Inc., Schaumburg, Illinois; 2 Pfizer, Inc., Groton, CT

## Abstract

**Background:**

Typical gram-positive organisms causing bloodstream infections (BSI) include *Staphylococcus aureus* (methicillin-susceptible [MSSA] and -nonsusceptible [MRSA]), coagulase negative staphylococci, *Streptococcus pneumoniae* and beta hemolytic streptococci. The parenteral cephem ceftaroline fosamil is approved for treatment of patients with community-acquired bacterial pneumonia caused by *S. pneumoniae* (including cases with concurrent bacteremia), MSSA, *Haemophilus influenzae*, and some species of Enterobacterales. Limited data have been published on the *in vitro* activity of ceftaroline against recent gram-positive clinical isolates known to be frequent bacterial causes of blood stream infections.

**Methods:**

Standard CLSI broth microdilution MIC determinations (M07) were performed with ceftaroline and comparator agents. MICs were interpreted using 2021 CLSI MIC breakpoints. Clinically relevant, non-duplicate, isolates cultured from blood by clinical laboratories in 2012-2019 were tested by the ATLAS (Antimicrobial Testing Leadership and Surveillance) program central laboratory (IHMA, Inc., Schaumburg, IL, USA). In total, 21,967 non-duplicate isolates of *S. aureus*, *S. epidermidis*, *S. pneumoniae* and beta hemolytic streptococci from BSI collected between 2012 and 2019 were tested. Isolates came from (n/%): Asia/South Pacific (2,970/13.5%), Europe (13,691/62.3%), Latin America (2,824/12.9%), MidEast/Africa (1,498/6.8%), and North America (Canada only) (984/4.5%).

**Results:**

Ceftaroline and comparator agent activities are summarized in the following table.

Results Table

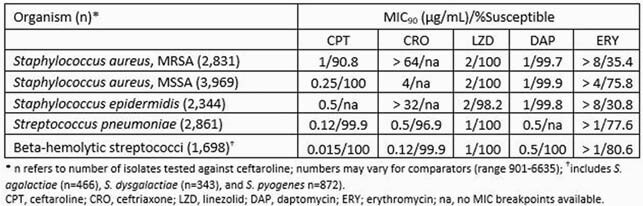

**Conclusion:**

Greater than 99% of *S. pneumoniae,* beta-hemolytic streptococci and MSSA isolates included in a 2012-2019 collection of gram-positive blood stream pathogens were susceptible to ceftaroline. 90.8% of MRSA were susceptible, and 9.1% isolates categorized as susceptible-dose dependent (MIC, 2-4 µg/mL); four isolates (two from Thailand and one each from China and S. Korea) were resistant to ceftaroline (MIC >4 µg/mL). The ceftaroline MIC_90_ for *S. epidermidis* was 0.5 µg/mL, with 97.7% of MICs ≤1 µg/mL. Ceftaroline continues to demonstrate potent *in vitro* activity against clinically relevant pathogens associated with BSI.

**Disclosures:**

**Meredith Hackel, PhD MPH**, **IHMA** (Employee)**Pfizer, Inc.** (Independent Contractor) **Gregory Stone, PhD**, **AztraZeneca** (Shareholder, Former Employee)**Pfizer, Inc.** (Employee) **Daniel F. Sahm, PhD**, **IHMA** (Employee)**Pfizer, Inc.** (Independent Contractor)

